# Crossmodal Interactions during Affective Picture Processing

**DOI:** 10.1371/journal.pone.0089858

**Published:** 2014-02-27

**Authors:** Vera Ferrari, Serena Mastria, Nicola Bruno

**Affiliations:** 1 Department of Neuroscience, University of Parma, Parma, Italy; 2 Department of Psychology, University of Bologna, Bologna, Italy; University of Bath, United Kingdom

## Abstract

"Natural" crossmodal correspondences, such as the spontaneous tendency to associate high pitches with high spatial locations, are often hypothesized to occur preattentively and independently of task instructions (top-down attention). Here, we investigate bottom-up attentional engagement by using emotional scenes that are known to naturally and reflexively engage attentional resources. We presented emotional (pleasant and unpleasant) or neutral pictures either below or above a fixation cross, while participants were required to discriminate between a high or a low pitch tone (experiment 1). Results showed that despite a robust crossmodal attentional capture of task-irrelevant emotional pictures, the general advantage in classifying the tones for congruent over incongruent visual-auditory stimuli was similar for emotional and neutral pictures. On the other hand, when picture position was task-relevant (experiment 2), task-irrelevant tones did not interact with pictures with regard to their combination of pitch and visual vertical spatial position, but instead they were effective in minimizing the interference effect of emotional picture processing on the ongoing task. These results provide constraints on our current understanding of natural crossmodal correspondences.

## Introduction

Natural perception is multisensory. We apprehend the world around us using signals from various sensory channels, and the input from these channels is fused into unitary percepts by sophisticated mechanisms. Perhaps one of the most intriguing of such mechanisms involves the spontaneous experience of correspondences between seemingly unrelated signals carried by different sensory channels. For instance, we naturally tend to match high-pitched sounds to bright, small, angular objects at higher locations in space, and low-pitched sounds to darker, bigger, rounder objects at lower locations [1]–[9]. These spontaneous or “natural” crossmodal correspondences (sometimes also called synesthetic correspondences or crossmodal similarities) are relevant for understanding the processes involved in multisensory integration.

Although studies of natural crossmodal mappings have a long history [2], [4], [10], the nature of perceptual processes mediating these phenomena is still largely unknown. An important question with this regard is whether such mappings are automatic or require attentional engagement. Given evidence for bidirectional influences between attention and multisensory perception [11]–[13], and given the seemingly arbitrary connection between the stimulus dimensions involved in natural correspondences, one might expect that attention should be necessary. Recent evidence suggests that the role of attention may be important to enhance the perceptual salience of the stimulus dimensions involved in the crossmodal correspondence [14].

However, other findings [15]–[16] are more consistent with preattentive processing of crossmodal mappings between auditory pitch and visual features.

In general, there are two modes by which attention is implicated in stimulus processing: First, in a top-down or goal-directed fashion as a function of instructions or task. Most of the research on crossmodal interactions has manipulated attention in this manner, that is by instructing participants to direct attention towards or away from stimulus features involved in the crossmodal mapping [1]–[2], [4], [15].

An alternative way to engage attention is instead by virtue of intrinsic relevance [17]–[18], in which attention is spontaneously oriented towards motivationally relevant stimuli in a bottom-up or stimulus-driven manner. In this project, we investigate attentional engagement in this second sense by using emotional natural scenes as visual cues as we are specifically interested in studying the impact of these meaningful stimuli in the processing of crossmodal correspondences, with the goal to assess basic multisensory processes in a context that resembles the incidental processing of the many-faceted stimuli encountered in daily life.

Pictures of pleasant and unpleasant natural scenes are known to engage attentional resources naturally and reflexively [17]–[22]. This is often called “motivated attention” and it is a specific type of bottom-up attention. The activation of motivational systems is believed to initiate a cascade of sensory and motor processes, including enhanced attention, information gathering, and preparation for action, that have evolved to assist in selecting appropriate survival behaviors [18]–[19]. Several findings demonstrate that when attentional resources are limited, emotionally arousing stimuli are perceived more efficiently than neutral stimuli [23]–[27]. Neuroimaging data also suggest that the visual system is engaged in more extensive and sustained processing when viewing emotional pictures than when processing less arousing stimuli [28]–[31]. Moreover, some data suggest that the enhanced processing prompted by affective stimuli may also interfere with the processing of concurrent stimuli close in space and time [32]–[33]. This is consistent with the hypothesis that emotional cues capture attention due to their evolutionary relevance, and that consequently competing non-motivationally relevant stimuli receive lesser amount of perceptual processing.

Here we investigated whether the viewing of task-irrelevant emotional scenes modulated visuo-acoustic natural correspondence effects using a modified version of the traditional speeded classification task [1]–[10], [15], [34]–[35]. Specifically, we replaced visual gratings in earlier studies with pictures of natural scenes that varied in affective content. Similarly to previous studies, our paradigm involved brief presentations of visuo-acoustic stimuli that varied in their combination of auditory pitch and visual vertical spatial position. Based on earlier results, one might predict that responses should be faster for congruent visuo-acoustic pairs (high pitches with top pictures, and low pitches with bottom pictures) and slower for incongruent ones (low pitches with top pictures, and high pitches with bottom pictures), in comparison to unisensory targets.

If the featural correspondences between basic auditory and visual features rely on the amount of attention available to somehow consciously process these features, we should observe that crossmodal correspondences are compromised by viewing motivationally relevant visual pictures. If crossmodal correspondences are truly automatic and preattentive one would instead predict that the magnitude of the crossmodal congruency effects remains largely independent of picture emotional content.

To test whether the arousal dimension of emotions modulates crossmodal congruence effects, we compared pictures with emotional content to neutral pictures. To test whether the effects depend on the valence dimension of emotions, we compared pleasant and unpleasant pictures. Finally, to examine whether the impact of emotional visual processing on crossmodal correspondences depends on which sensory modality is task-relevant, we compared responses whereby participants' attention was directed to the auditory channel (experiment 1) with those whereby it was directed to the visual channel (experiment 2).

## Experiment 1

Participant performed a speeded classification task on auditory stimuli (is it a high or low tone?). In most trials, the tone was coupled with a picture that was presented either above or below the screen center. The picture was either emotionally relevant or neutral. We were interested in determining whether the position of the picture affects the processing of pitch, in analogy to what happens with simpler stimuli [15], and whether this effect was modulated by the emotional relevance of the picture.

### Methods

#### Participants

Twenty-two right-handed students (12 women) from the University of Parma participated. All were naive as to the purpose of the study and had normal or corrected-to-normal visual acuity.

#### Ethics Statement

All participants were 18 years or older at the time of the study. Prior to participating, all were informed concerning the potentially disturbing content of some stimuli and signed a written informed consent in accordance with the ethical standards laid down in the 1964 Declaration of Helsinki.

The research was conducted in accordance with the ethical standards of the Italian Board of Psychologists (see http://www.psy.it/codice_deontologico.html) and of the Italian Psychological Society (AIP, see http://www.aipass.org/node/26). Given that the experiment did not involve clinical tests, use of pharmaceuticals or medical equipment, did not involve the use of deception or involve participant discomfort in any other way, approval of Ethics Committee for Clinical Research of the University of Parma was deemed unnecessary, in accordance with the regulation of the local ethical committee of the University of Parma.

#### Stimuli and apparatus

The visual stimuli were color pictures of natural scenes selected from various sources, including the International Affective Picture System (IAPS; [36]; see File S1), and public domain pictures available on the Internet, consisting of 48 pleasant, 48 neutral and 48 unpleasant pictures. The mean (and SEM) valence scores of the selected pictures in undergraduate Italian samples [27] were 6.73 (.71), 5.76 (.34), 2.55 (1.10) for, respectively, pleasant, neutral, and unpleasant pictures in a 9-point scale, and these valence scores were all statistically different from each other, Fs(2,28)>42.4, ps<.001, η^2^>.75. The respective rated arousal scores were 4.14 (1.91), 2.51 (1.17), 5.23 (2.10) for pleasant, neutral, and unpleasant pictures, where pleasant and unpleasant pictures were rated as more arousing than neutral pictures F(1,14) = 37.4, p<.001, η^2^ = .73, and did not differ from each other F(1,14) = 3.2, p>.05.

Each picture subtended a visual angle of 6° (width) x 4° (height), at a constant viewing distance of 57 cm. A picture stimulus could be presented 2° either above or below a black central fixation cross (subtending a 1°×1° of visual angle). All pictures were presented in their original colors against a grey (80 cd/m^2^ ) background. The pictures were displayed on a 19-in. CRT monitor with a 100-Hz refresh rate, at a resolution of 800×600 pixels. The auditory stimuli were two tones of 1500 Hz (high pitch) and 1000 Hz (low pitch). We presented them for 120ms, with a rise and fall time of 10 ms, volume adjusted to 80 dB, using a set of Bose AE2 headphones for external noise reduction. Participants had their heads positioned in a chin and forehead rest. E-Prime software [37] was used to present the pictorial and acoustic stimuli and to record the behavioral responses. Response accuracy and latency on the speeded classification task were collected through a standard computer keyboard.

#### Design and Procedure

At the beginning of the experiment, we ran a tone discrimination training session (10 trials) to familiarize participants with the two tones used in the experiment. During this preliminary phase, we informed participants that pictures will also be presented in most of the trials and that these will be simultaneous with the tone. We explicitly told participants that these pictures were irrelevant to the task and therefore had to be ignored. Each trial began with a fixation cross that was presented for 500 ms at the center of the screen. This was always followed by the presentation of one of two tones, randomly varying in pitch (1000 or 1500 Hz). Tones either occurred alone (*auditory only* condition) or were paired with one picture (*visual-auditory* condition). Tones and pictures had the same onset and duration (120 ms). The picture appeared either below or above the fixation cross, and was randomly paired with a low or a high pitch tone, resulting in congruent (high picture position and high pitch, low picture position and low pitch) or incongruent (high position but low pitch, low position but high pitch) crossmodal correspondences.

Over the whole experiment, 672 trials were presented, divided into 4 blocks of 168 trials each. Within each block, 24 trials consisted of tone presentation only (equal number of high and low pitch), whereas in the remaining 144 trials an equal number of pleasant, unpleasant and neutral pictures were presented either above or below fixation, and were paired with either a high or a low-pitch tone (12 trials per condition in each of the four blocks). The same picture stimulus appeared 4 times (one in each block), such that across the whole experiment, each unique visual stimulus was associated with all the four experimental conditions.

Participants performed a speeded auditory classification task. In each trial, they fixated the central cross, heard the tone, and classified it as either high or low by pressing appropriately marked keys, as quickly and accurately as possible. For half of the participants, the high key was on the right and the low key on the left. For the other half of participants, this mapping was reversed. There was a variable interval of 1800–2000 ms before the start of the next trial.

#### Data Analysis

Reaction Times (RTs) were analyzed only for trials associated to correct responses. Responses were scored as correct if the appropriate response latencies were not shorter or longer than the mean (within each subject and each condition) ± 3 times the standard deviation of the mean.

**Figure 1:**
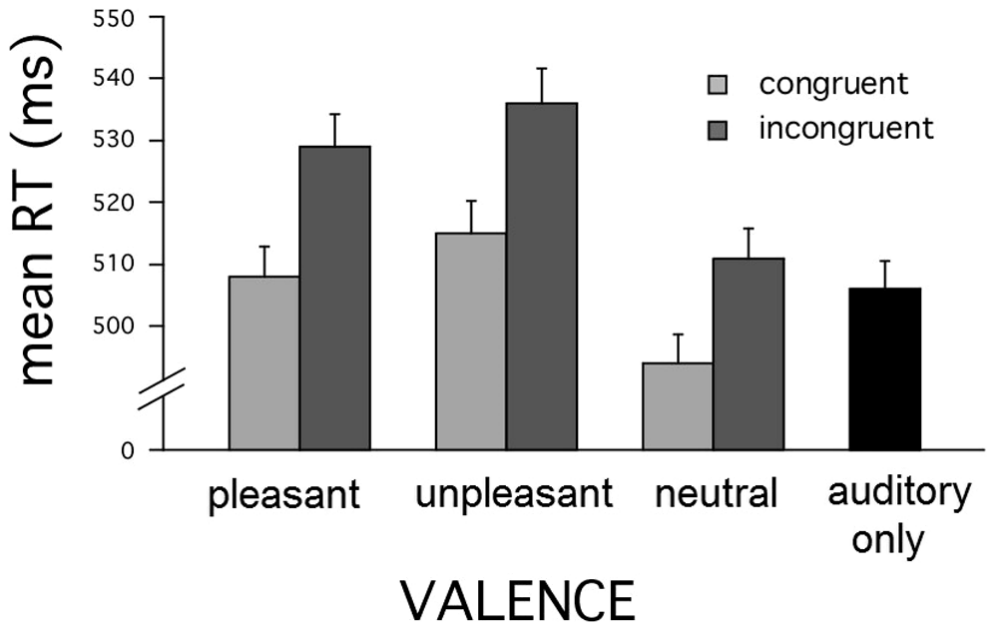
Overview of findings in Experiment 1 (auditory classification). Average response times as a function of emotional valence (pleasant, neutral, unpleasant pictures) in the visual scene and of crossmodal congruence. Error bars: 1 SEM.

Significance tests were then performed using a repeated-measure analysis of variance (ANOVA), with the factor of congruence (two levels: visual-auditory congruence or incongruence) and visual emotional valence (three levels: pleasant, unpleasant, neutral). To provide a baseline reference for evaluating reaction times, we also computed summary statistics for trials in the auditory-only condition. Raw RT means for all conditions (along with standard errors) are presented in Figure 1.

### Results

As shown in figure 1, we observed two clear main effects. The first was a crossmodal congruence effect, F(1,21) = 22.8, p<.0001, η^2^ = .52, indicating that participants were generally faster in classifying pitches in congruent, compared to incongruent, trials. The second was the effect of picture valence, F(2,42) = 4.7, p<.05, η^2^ = .18, indicating that reaction times to the tones were affected by the affective content of task-irrelevant visual scenes. Post-hoc pairwise tests indicated that RTs were slower with both pleasant and unpleasant pictures in comparison to neutral ones, F(1,21) = 16.9, p<.0001, η^2^ = .44, F(1,21) = 4.8,p<.05, η^2^ = .19, respectively, whereas RTs associated with viewing pleasant or unpleasant pictures did not differ, p>0.9. Finally, and crucially for the research question that motivates this work, the approximate 20ms advantage of congruent over incongruent auditory-visual trials was nearly constant across all valence contents. Accordingly, we did not observe a significant interaction between congruence and picture valence (F = 0.2, p >0.8). In a second analysis, we performed a second test of crossmodal congruence by comparing emotionally neutral congruent and incongruent visual-auditory trials to auditory-only trials by means of a one-way repeated measures ANOVA. This analysis also confirmed a significant effect, F(2,42) = 7.2, p<0.005, η^2^ = .25 and post-hoc pairwise comparisons indicated that the effect involved significant differences between the auditory-only and the visual-auditory congruent trials, F(1,21) = 14.7, p<.005, η^2^ = .42, as well as between the visual-auditory congruent and incongruent trials, F(1,21) = 8.7, p<.01, η^2^ = .29, but not between the auditory-only and the visual-auditory incongruent ones, F<1. Overall accuracy in the acoustic classification task was high (average 97.6%). However, participants were slightly more accurate for congruent than for incongruent combinations of visuo-acoustic stimuli, F(1,21) = 4.6, p<.05, η^2^ = .18.

### Discussion

Replicating previous studies [15], [35], experiment 1 found a clear crossmodal congruence effect on reaction times, with faster responses for congruent, compared to incongruent, combinations of visuo-acoustic features. However, at least in the context of the present experiment, the magnitude of the crossmodal congruency effects was not modulated by the emotional content of the visual stimuli. The advantage in classifying the tones for congruent over incongruent visual-auditory stimuli was just as large with the emotionally relevant visual stimuli and with the neutral visual stimuli, and did not differ between pleasant and unpleasant picture content. These results support the idea that crossmodal mappings reflect intrinsic correspondences at the sensory level and are relatively immune to motivated attention. We did observe, however, a robust crossmodal attentional interaction in that presenting pleasant and unpleasant visual pictures yielded generally slower RTs in all conditions in comparison to neutral pictures, indicating that task-irrelevant emotional visual scenes were effective in capturing attention [17], [20], [38].

## Experiment 2

Previous studies on crossmodal correspondences have reported similar congruence effects across task modalities. For instance, congruence effects have been reported both when participants responded to tones and when they responded to visual stimuli [15]. Based on these findings, we should expect to replicate the results of experiment 1 if the attended channel is visual and the tones varying in pitch now become irrelevant. Thus, in experiment 2, participants performed a speeded classification task on visual stimuli (was the picture presented above or below fixation?). In two thirds of the trials, the picture was coupled with a tone that could be high or low in pitch. As in Experiment 1, pictures were either neutral or emotionally relevant. We were interested in determining whether the pitch of the tone affects the processing of picture position, again in analogy to what happens with tones coupled with simpler visual stimuli, and whether this effect is modulated by the emotional content of the picture.

### Methods

#### Participants

Twenty-two right-handed students (13 women) from the University of Parma participated. All were naive and had normal or corrected-to-normal acuity. As in the previous experiment, prior to participation all were informed concerning the potentially disturbing content of some experimental stimuli and gave their consent in accordance with Helsinki standards.

#### Stimuli and apparatus

The experimental design mirrored that of the previous experiment as in a portion of trials we presented only visual stimuli with no tones (*visual-only* condition) and compared those to trials that included the presentation of the tones (*auditory-visual* condition). For each valence, 24 pictures were added to the stimulus set used in experiment 1, such that there was a total of 72 pleasant, 72 unpleasant, and 72 neutral pictures. For each valence category, 24 were presented in the visual only condition, 24 in the congruent auditory-visual condition, and 24 in the incongruent one. Three sets were constructed that varied, across participants, the specific pictures presented in the three experimental conditions (unisensory, crossmodal congruent and incongruent). In all other respects, stimuli and apparatus were the same as those of Experiment 1.

#### Procedure

It was similar to that of experiment 1. At the beginning there was a practice phase of 10 trials, in which we presented neutral pictures and familiarized participants with the visual classification task. Participants were informed that they would hear tones at the same time as they saw the pictures, but that these tones were irrelevant to the task and therefore had to be ignored. Next, we initiated the test phase, which consisted of 720 trials divided into 4 blocks of 180 each. Participants performed a visual speeded classification task. In each trial, they fixated the central cross, saw the picture, and classified it as either high or low by pressing appropriately marked keys, as quickly and accurately as possible. Also similar to the previous study we inserted a variable inter-trial interval of 1800–2000 ms.

#### Data Analysis

Data analysis was identical to that of experiment 1. Unlike experiment 1, however, the main analysis included also the unimodal condition (i.e. visual only), as the unimodal trials also varied along the valence dimension. Thus, RTs data were analyzed in a two-way ANOVA, using crossmodal congruence (visual unisensory, crossmodal congruent or incongruent) and picture valence (pleasant, unpleasant, and neutral) as factors.

### Results

**Figure 2:**
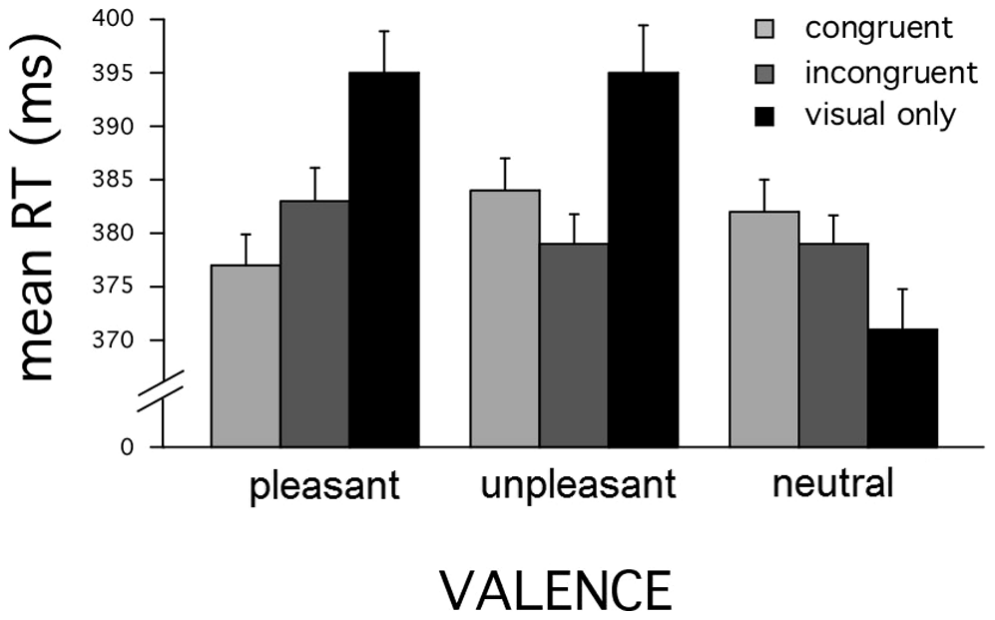
Overview of findings in Experiment 2 (visual classification). Average response times as a function of emotional valence (pleasant, neutral, unpleasant pictures) in the visual scene and of crossmodal congruence. Error bars: 1 SEM.

Figure 2 shows mean RTs in the visual speeded classification task. A 3 (valence) X 3 (congruence) repeated-measures ANOVA revealed significant main effects of valence, F(2,42) = 6.9, p<0.01,η^2^ = .25, and congruence, F(2,42) = 9, p <0.001, η^2^ = .3, and as well as a significant two-way interaction, F(4,84) = 5.4, p<0.01, η^2^ = .22. Similarly to experiment 1, the valence effect was due to slower RTs for pleasant and unpleasant pictures, compared to neutral contents (p<.05, p<.005,η^2^>13, respectively) with no difference between pleasant and unpleasant pictures, F<1. Post-hoc pairwise comparisons indicated that the congruence effect was due to the average increase of RT's in the visual only condition, relative to both congruent (p<0.01) and incongruent (p<0.005) trials, and, which did not differ (p>0.6). Moreover, the interaction primarily indicates that the RT slowdowns during emotional picture viewing (both pleasant and unpleasant) were reduced by the tone, regardless of the cross-modal congruence. Interestingly, this response acceleration prompted by the tone was such that the differences in RTs between emotional (pleasant and unpleasant) and neutral pictures clearly decreased and were not statistically reliable for both congruent and incongruent conditions (ps>.05).

The overall accuracy in the visual classification task was quite high (average 96 %), and was not reliably affected by the experimental manipulations.

### Discussion

These results confirm that, as we observed in the first experiment, picture emotional content did not modulate crossmodal correspondences. However, the data showed no hint of the congruence effect that we observed in the first experiment. In fact, independent of picture valence, average times were approximately the same for congruent and incongruent audiovisual pairings. On the other hand, when pictures were not accompanied by a sound, emotionally relevant visual stimuli captured attention and slowed down visual classification responses in comparison to emotionally neutral stimuli. Thus, as in the first experiment we observed an emotional capture effect. Interestingly, emotional capture was neutralized when the visual stimuli were accompanied by a tone, irrespective of its potential congruence with the visual location. Thus, in this experiment tones did not interact with pictures with regard to their pitch and visual vertical spatial position. Instead, they interacted with pictures with regard to the way that participants were affected by emotional content.

## Discussion

A critical question in the study of natural crossmodal correspondences is whether such multisensory interactions can be affected by higher level attentional factors [14]–[15], [39]–[40]. The present study provided an initial exploration of the impact of motivated attention, as engaged by stimulus emotional content, on crossmodal mapping of auditory pitch and visual elevation.

Results indicate that the crossmodal congruence effect is robust with an auditory classification task whereby tones are paired with irrelevant visual emotional scenes (experiment 1). In this study, and consistent with previous reports, we observed faster responses to tones for congruent, compared to incongruent, combinations of picture position and tone pitch. In addition, and crucially for the purpose of the present study, pleasant, unpleasant, and neutral pictures showed similar congruence effects, indicating that visual emotional content did not affect the extent to which interactions occurred. At the same time, emotional content did have a clear multisensory effect on the auditory classification performance, as responses to the tones were slower during viewing of pleasant and unpleasant pictures, compared to neutral ones. We can therefore rule out that the emotional content of our stimuli was not efficient to engage attention or to affect multisensory interactions.

However, results also indicate that the crossmodal congruence effect is not equally robust with a visual classification task whereby pictures are paired with irrelevant sounds (experiment 2). In this second study, we found no evidence of crossmodal congruence effects, suggesting that the task-irrelevant tones did not interact with the processing of picture position. The observed results instead suggest that tones prompted faster behavioural responses compared to when the picture was presented in isolation, and that this occurred specifically while viewing emotional pictures (both pleasant and unpleasant), as no change in response speed was observed for neutral pictures. In the visual unisensory trials, conversely, emotional pictures slowed down the responses exhibiting the typical attentional capture effect that in experiment 1 was evident for both congruent and incongruent combinations of visuo-auditory stimuli.

In sum, the results of experiment 1 may be consistent with previous studies that reported no effect of attention on cross-modal speeded classification tasks [15], supporting the hypothesis that features in different sensory channels interact preattentively at an early stage of stimulus processing, regardless of stimulus relevance. On the other hand, the results of experiment 2 suggest that crossmodal interactions may depend on the allocation of endogenous attention in certain conditions, especially when intrinsic stimulus salience is unbalanced across sensory modalities, (i.e. meaningful pictures compared to repetitive tones). Indeed in our experiment, behavioral effects of crossmodal correspondences occurred only when high and low tones were made salient by means of the instructions (task-relevant), contrariwise task-irrelevant tones did not interact with pictures with regard to their combination of pitch and visual vertical spatial position, possibly because of the attentional bias towards the visual information.

Reports of differences in the strength of audiovisual interactions, with generally stronger effects on pitch than on visual dimensions, are not new. At least two previous studies reported stronger effects of vision on audition in comparison to audition on vision [7], [15]. These studies suggested that the asymmetry may be due to slower RT’s during auditory, compared to visual, classification, allowing more time for the congruency to affect responses. Although an overall difference in RTs was also observed in our experiments, with faster RTs for the visual in comparison to the auditory task, a further potential cause for the asymmetry may have to do with the stimulus properties themselves. Indeed, it has previously been shown that if input in one modality is presented at a higher intensity or has a better quality of information than the input in another modality, the integration process may be biased by the more salient stimulus [35]. In experiment 2, cross-modal interactions may have been more biased towards vision because visual stimuli were meaningful pictures that varied across trials and because picture position was task-relevant. Auditory information was instead repetitive and irrelevant to the task. On the other hand, previous studies [15] have typically used relatively simple material. This material was motivationally neutral and highly repetitive over trials, such that the impact of intrinsic stimulus properties from either sensory modality was very low. These conditions may have allowed multisensory interactions to occur regardless of which modality was task-relevant. Additional studies, conducted in our laboratory to compare neutral natural scenes with Gabor gratings, yielded support for the above interpretation (see File S2).

Taken together, these findings are consistent with recent proposals [14], [39]–[40] that points towards a critical role of attention in order to make the dimensions on which the crossmodal correspondence operates perceptually salient to the participant. Once those dimensions have been made salient, the crossmodal correspondence might then operate in an automatic manner, and this could perhaps explain why there was no interference of emotional picture content on the crossmodal interaction in the auditory task (experiment 1). This interpretation is also in line with recent models of automaticity that overcome the classical dichotomy of top-down or bottom-up control of attention [41]–[42], as they propose that automatic (or unconscious) processing depends on higher-level, top-down factors such as attention, intentions, and task sets that orchestrate the processing streams toward greater optimization of task performance [43]–[45].

Despite the lack of crossmodal congruency effect in the visual task, we did however observe a striking evidence of an emotion-specific multisensory effect of task-irrelevant auditory stimuli. The typical interference effect of emotional picture processing on the primary task was indeed neutralized when the visual stimuli were accompanied by the task-irrelevant tone, irrespective of its potential congruence with visual location. This lack of affective modulation on task performance is consistent with some research showing that the attentional capture prompted by task-irrelevant emotional pictures may be affected by task-demands [46]–[49]. It has repeatedly been found that responses in reaction time tasks are shorter when a salient but task-irrelevant *accessory stimulus* (AS) presented in another perceptual modality accompanies the imperative stimulus, compared to when the imperative stimulus is presented alone [50]–[53]. However, at this stage it is hard to know whether the facilitatory effect of an AS on participant's performance was specific for emotional pictures or was rather a more general automatic alerting effect on motor execution processes, which did not emerge during the viewing of neutral pictures as the response speed was already as fast as it could be made (i.e. floor effect). Event-related potential studies, along with others psychophysiological methods, are well suited for testing these hypotheses.

## Supporting Information

File S1 **IAPS codes.**
(DOC)

File S2 **Experiment 3: Impact of stimulus material (Gabor patches vs pictures of natural scenes) on the magnitude of cross modal congruence effects.**
(DOC)
