# Supplementary material for: Crossmodal Interactions during Affective Picture Processing
Source: PLoS One. 2014 Feb 27;9(2):e89858. doi: 10.1371/journal.pone.0089858 (PMC3937419; doi:10.1371/journal.pone.0089858)
Supplement: File S1 — IAPS codes. (DOC) [file pone.0089858.s001.doc]

The codes for the IAPS pictures included in the stimulus set used in the current research project (experiment 1 & 2) were as follows: Neutral (0201, 0204, 0205, 0207, 0208, 0210, 0211, 0214, 02631, 2002, 2019, 2102, 2104, 2191, 2305, 2359, 2372, 2374, 2377, 2382, 2383, 2384, 2390, 2411, 2435, 2488, 2489, 2490, 2495, 2513, 2595, 2635, 2749, 5410, 5455, 5471, 5520, 7009, 7030, 7032, 7038, 7044,7234, 7493, 8118, 8311, 8312, 9927); Unpleasant (3000, 3001, 3010, 3015, 3016, 3030, 3051, 3053, 3060, 3063, 3064, 3069, 3071, 3080, 3100, 3101, 3103, 3110, 3120, 3130, 3140, 3150, 3160, 3168, 3170, 3180, 3181, 3185, 3191, 3195, 3213, 3225, 3261, 3266, 3400, 6200, 6210, 6212, 6213, 6220, 6231, 6242, 6243, 6244, 6250, 6260, 6300, 6312, 6313, 6314, 6315, 6350, 6360, 6410, 6510, 6520, 6530, 6540, 6550, 6560, 6563, 6570; Pleasant (0461, 0463, 0464, 2530, 2550, 4597, 4604, 4607, 4611, 4612, 4616, 4619, 4623, 4624, 4625, 4626, 4640, 4641, 4643, 4645, 4647, 4649, 4650, 4651, 4652, 4653, 4656, 4658, 4659, 4660, 4664, 4666, 4669, 4670, 4672, 4680, 4687, 4689, 4690, 4692, 4693, 4694, 4695, 4697, 4698, 4800, 4810).
