# Supplementary material for: Crossmodal Interactions during Affective Picture Processing
Source: PLoS One. 2014 Feb 27;9(2):e89858. doi: 10.1371/journal.pone.0089858 (PMC3937419; doi:10.1371/journal.pone.0089858)
Supplement: File S2 — Experiment 3: Impact of stimulus material (Gabor patches vs pictures of natural scenes) on the magnitude of cross modal congruence effects. (DOC) [file pone.0089858.s002.doc]

The procedure was similar to that of experiment 1 and 2 in the manuscript. Two groups of 14 participants each took part in the study. One group performed an auditory speeded classification task, whereas the other performed a visual speeded classification task. In each task, two blocks of 240 stimuli were presented (equal number of unisensory and multisensory congruent or incongruent combinations of visuo-acoustic stimuli). Pictures of natural scenes or Gabor patches were presented as visual stimuli in separate blocks. The order of presentation of stimulus material (pictures vs. Gabors) was counterbalanced across participants. Congruent, compared to incongruent, combinations of Gabors and auditory stimuli showed faster responses in both tasks (Auditory: 529ms ± 29 vs. 567ms ±35; Visual: 545ms ± 31 vs. 556ms ± 32). The pattern of RT modulation was similar when Gabors were replaced with pictures of natural scenes, although less pronounced, especially in the visual classification task (Auditory: 385ms ± 18 vs. 402ms ±21; Visual: 368ms ± 17 vs. 372ms ±18). A 2 (Task: Auditory, Visual) X 2 (Stimulus material: Picture, Gabor) X 3 (congruence: Congruent, Incongruent, Unimodal) ANOVA confirmed statistically significant effects of congruence F(2,52)=11.42, p<.0001, as well as the interaction Stimulus material X Congruence F(2,52)=5.86, p<.01, indicating a smaller congruence effect for pictures compared to Gabor patches in both tasks. Responses to unisensory trials were similar between pictures and Gabors (F<1). Taken together, these results suggest that the type of stimulus material used to study crossmodal correspondences may affect the magnitude of the congruence effect: More complex or relevant stimuli presented to one sensory channel may reflexively attract attention at the expense of concurrent information provided through other sensory channels, thus preventing the natural feature correspondence.
